# Supplementary material for: Cuproptosis-Related MiR-21-5p/FDX1 Axis in Clear Cell Renal Cell Carcinoma and Its Potential Impact on Tumor Microenvironment
Source: Cells. 2022 Dec 31;12(1):173. doi: 10.3390/cells12010173 (PMC9818076; doi:10.3390/cells12010173)
Supplement: Supplementary file 1 [file cells-12-00173-s001.zip › cells-2041680-supplementary.pdf]

Table S1. The clinicopathological characteristics of ccRCC patients in micro-array

Patient ID	Gender	Age	Tumor size (cm)	Stage	Grade	Survival time(m)	Adjacent tissue
1#	male	79	5	T1b	2	87	Yes*
2#	male	62	10x5x4	T2b	3	86	Yes*
3#	female	42	3	T1a	1	85	Yes*
4#	male	52	2.5x2x2	T1a	2	84	Yes*
5#	male	66	5.5x4.5x4	T1b	3	83	Yes*
6#	male	78	4x4x4.5	T1b	3	80	Yes*
7#	female	47	2.6x2.5x1.5	T1a	3	87	Yes*
8#	male	30	5	T1b	2	87	Yes*
9#	male	49	3.2x2.5x2.2	T1a	2	86	Yes*
10#	male	46	2.6X3.2X3.7	T1a	1	84	Yes*
11#	male	49	3x3x2.5	T1a	2	81	Yes
12#	female	75	4	T1a	2	79	Yes
13#	female	48	4.8x4x4	T2a	3	79	Yes
14#	male	43	3.5	T1a	1	77	Yes
15#	female	56	4.2X5.2X7.2	T3a	3	77	Yes
16#	male	57	3	T2a	1	75	Yes
17#	female	51	3x2x1.4	T1a	2	75	Yes
18#	male	63	7.5x7x5.5	T2a	3	75	Yes
19#	male	58	4	T3a	3	73	Yes
20#	male	63	8x8x7	T2a	2	72	Yes
21#	male	65	6x4.5x4	T2b	3	68	Yes
22#	female	55	10x7x6	T2b	3	67	Yes
23#	male	53	2.2x2.2	T1a	1	66	Yes
24#	male	51	5x4.5x4	T3a	3	66	Yes
25#	female	53	3	T1a	1	66	Yes
26#	male	65	4	T1a	2	64	Yes
27#	female	31	3x2.5x2	T1a	1	63	Yes
28#	male	59	3x3x1.2	T1a	1	62	NO
29#	female	41	3.5x3x2.5	T1a	1	62	NO
30#	male	57	3.5x3.5x3.3	T1a	1	62	NO
31#	female	72	2.5	T1a	1	62	NO
32#	female	64	4x3x2.5	T1a	1	62	NO
33#	male	75	7.5x6.5x5	T2a	3	61	NO
34#	male	57	5x5x3	T1b	1	61	NO
35#	female	68	7x6.5x5	T1b	1		NO
36#	female	39	10x10x5	T2a	3		NO
37#	male	71	5x4.2x4	T1b	3		NO
38#	male	76	5x4.5x2.5	T1b	2		NO
39#	female	62	6.3	T1b	2		NO

40#	female	51	5x4x2.5	T1b	3	NO
41#	female	50	2x1.5x1	T1b	2	NO
42#	male	51	4.5x4x3.5	T1b	1	NO
43#	male	68	5x3.5	T1b	2	NO
44#	male	65	6x6x4.5	T1b	2	NO
45#	male	45	9	T3a	3	NO
46#	male	64	6.5x5x5	T3a	1	NO
47#	male	70	7x5x5	T1b	2	NO
48#	female	77	7x6.5x5.5	T1b	3	NO
49#	male	68	4x4x3	T1a	2	NO
50#	male	60	6x5x4	T1b	3	NO
51#	male	68	4	T1a	3	NO
52#	male	55	4.5X3.6			NO
53#	male	72	7.2X6.3X5.5			NO
54#	female	74	6.6			NO
55#	male	47	4.3X4.9			NO
56#	male	46	4.3X6.4X7.2			NO
57#	male	64	3.4X4.9X6.2			NO
58#	female	68	5.5			NO
59#	male	65	5.4X3.5X6.2			NO
60#	female	73	5X3.5X4.5			NO
61#	female	68	6.8X7X5			NO
62#	male	46	5X4.4			NO

Notes: *marked ccRCC patient as well as its adjacent kidney tissue was used for western blotting analysis in Figure 4A.

Table S2. The associations between the expression levels of cuproptosis regulators and the clinicopathological characteristics of ccRCC in TCGA-KIRC

genes	FDX1	LIAS	LIPT1	DLD	DLAT	PDHB	MTF1	GLS	CDKN2A	PDHA1
alive	10.44	9.54	8.00	12.31	11.07	10.82	10.29	13.57	7.98	4.05
dead	10.25	9.42	7.97	12.07	10.65	10.71	9.99	13.51	8.31	4.02
P value	0.0003	0.0603	0.4816	0.0004	<0.0001	0.0084	<0.0001	0.0928	0.0095	0.3062
P.symbol	***	ns	ns	***	****	**	****	ns	**	ns
<=30	10.76	9.08	7.58	11.94	10.78	11.13	10.16	12.94	7.75	4.09
30-60	10.37	9.50	7.98	12.22	10.95	10.80	10.20	13.53	8.00	4.03
>=60	10.38	9.50	8.01	12.24	10.91	10.77	10.19	13.56	8.17	4.05
P value	0.6978	0.6403	0.5312	0.864	0.8744	0.621	0.9943	0.528	0.2243	0.9287
P.symbol	ns	ns	ns	ns	ns	ns	ns	ns	ns	ns
female	10.45	9.47	8.00	12.28	11.00	10.83	10.29	13.74	8.19	4.07
male	10.34	9.51	7.99	12.21	10.90	10.76	10.15	13.45	8.04	4.02
P value	0.1633	0.3121	0.8098	0.4744	0.2	0.334	0.0668	0.0027	0.401	0.1577
P.symbol	ns	ns	ns	ns	ns	ns	ns	**	ns	ns
1	9.99	9.46	7.64	11.92	10.94	10.60	10.42	13.39	8.01	3.91
2	10.43	9.58	8.04	12.28	11.04	10.81	10.30	13.51	7.78	4.04
3	10.37	9.49	7.99	12.23	10.91	10.78	10.11	13.60	8.25	4.06
4	10.27	9.27	7.89	12.12	10.68	10.76	10.08	13.53	8.57	4.00
P value	0.0681	0.0036	0.0502	0.3155	0.0369	0.7063	0.057	0.6245	<0.0001	0.5711
P.symbol	ns	**	ns	ns	*	ns	ns	ns	****	ns
1	10.47	9.61	8.02	12.37	11.12	10.89	10.36	13.60	7.94	4.07
2	10.27	9.55	8.09	12.16	10.94	10.73	10.02	13.44	8.02	4.08
3	10.33	9.35	7.94	12.06	10.69	10.63	10.03	13.49	8.28	3.98
4	10.25	9.35	7.92	12.13	10.71	10.75	10.06	13.54	8.29	4.03
P value	0.019	0.0001	0.2302	0.0035	<0.0001	0.0018	0.0003	0.5252	0.0153	0.2323
P.symbol	*	***	ns	**	****	**	***	ns	*	ns
1	10.46	9.61	8.03	12.37	11.12	10.89	10.36	13.60	7.96	4.07
2	10.25	9.51	8.04	12.10	10.83	10.72	9.94	13.47	8.07	4.07
3	10.31	9.33	7.93	12.08	10.70	10.65	10.05	13.50	8.22	3.98
4	10.24	9.49	7.87	12.31	11.01	10.99	10.28	13.46	9.08	4.13
P value	0.0273	0.0001	0.2362	0.0027	<0.0001	0.0008	0.0001	0.5417	0.0027	0.1993
P.symbol	*	***	ns	**	****	***	***	ns	**	ns
no	10.40	9.52	8.01	12.25	10.98	10.80	10.22	13.55	8.08	4.05
yes	10.23	9.35	7.92	12.12	10.69	10.72	10.05	13.55	8.15	4.01
P value	0.0279	0.0227	0.2009	0.1246	0.0018	0.0916	0.0357	0.9718	0.2921	0.2023
P.symbol	*	*	ns	ns	**	ns	*	ns	ns	ns
no	10.40	9.53	8.00	12.29	10.95	10.82	10.21	13.60	8.07	4.04
yes	10.07	9.14	7.87	12.11	10.55	10.66	10.07	13.49	8.07	4.00
unknown	10.37	9.49	7.99	12.18	10.93	10.76	10.19	13.50	8.11	4.04
P value	0.1587	0.0665	0.7219	0.2942	0.2685	0.4786	0.8354	0.4063	0.9314	0.9549
P.symbol	ns	ns	ns	ns	ns	ns	ns	ns	ns	ns

Supplementary Figure S1

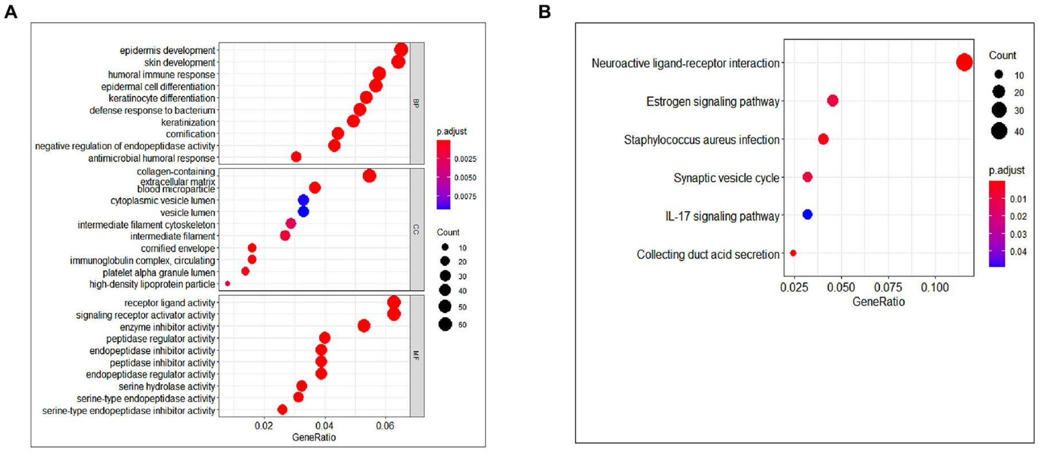

Figure S1: GO and KEGG analyses of CSS and CRS. (**A**) GO analysis of the differentially expressed genes (DEGs) between CSS and CRS. (**B**) KEGG analysis of differentially expressed genes (DEGs) between CSS and CRS.

Supplementary Figure S2

A

Univariate Cox regression analysis of cuproptosis regulators

ID	HR	HR. 95L	HR. 95H	pvalue
FDX1	0. 47545855	0. 328079296	0. 689043277	8. 58E-05
LIAS	0. 552012965	0. 374256239	0. 814197018	0. 002729912
LIPT1	0. 822168597	0. 527161043	1. 282266985	0. 387854286
DLD	0. 612325069	0. 468145508	0. 800909084	0. 000342746
DLAT	0. 467696903	0. 348943937	0. 626864004	3. 68E-07
PDHB	0. 637169624	0. 418139832	0. 970931488	0. 035973532
MTF1	0. 513761553	0. 368186895	0. 716893883	8. 93E-05
GLS	0. 925475631	0. 726917393	1. 178270259	0. 529636624
CDKN2A	1. 544693736	1. 251505762	1. 906566323	5. 14E-05
PDHA1	0. 806520646	0. 56536676	1. 150537312	0. 235499437

B

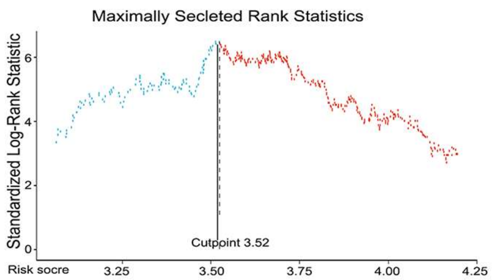

Figure S2: Univariate Cox regression analysis and cutoff point of risk score. (**A**) Univariate Cox regression analysis of the cuproptosis related regulators. (**B**) Cuproptosis risk score selection based on the analysis from the survcutpoint in the survminer R package.
